# Is Schizotypy *per se* a Suitable Endophenotype of Schizophrenia? – Do Not Forget to Distinguish Positive from Negative Facets

**DOI:** 10.3389/fpsyt.2015.00143

**Published:** 2015-10-23

**Authors:** Phillip Grant

**Affiliations:** ^1^Biological Psychology and Individual Differences, Department of Psychology, Justus-Liebig-University Giessen, Giessen, Germany

**Keywords:** schizotypy, psychosis, schizophrenia, endophenotypes, psychosis proneness, genetic predisposition to disease

In 2003, Gottesman and Gould advocated using the endophenotype concept in psychiatry genetics; describing it as a measurable component “along the pathway between disease and distal genotype” (p. 636). Especially in schizophrenia research, the idea of endophenotypes has gathered a wide following. Meehl’s definition of schizotaxia [Ref. (1), p. 829], as “an ‘integrative neural defect’ as the only direct phenotypic consequence produced by the genetic mutation” and “This neural integrative defect […] is all that can properly be spoken of as inherited. The imposition of a social learning history upon schizo*taxic* individuals results in a personality organization which I shall call, following Rado, the schizo*type*” (p. 830; my emphasis) reminds clearly of the aforementioned endophenotype description.

Although there are a number of ongoing debates regarding the nature of schizotypy, its distribution throughout the population and its relation with schizophrenia, a few elements may be considered “agreed upon”: (1) schizotypy is a latent trait (partly) harboring individual risk for schizophrenia (2). (2) Schizotypy is (partly) influenced by genes considered relevant for schizophrenia; q.v. (3). (3) Schizotypic variance is best explained through gene–environment interactions of a kind also relevant for schizophrenia (4). (4) Schizotypy shares a factor-structure with symptoms of schizophrenia; with the most commonly replicated factors being the positive, negative, and disorganized facets (5, 6). It therefore appears that schizo*typy* and schizo*phrenia* qualitatively share various similarities, albeit in a quantitatively different fashion.

It is not surprising that a number of measures considered endophenotypes of schizo*phrenia* [q.v. (7)] have also been shown to be associated with schizo*typy* (8). The question at hand (whether schizotypy *per se* may qualify as a suitable endophenotype for schizophrenia) has, to my knowledge, not been answered as yet. I have briefly argued thusly elsewhere (9) and will, here, further elaborate on this and continue discussing the implications of the debated link between schizophrenia-genetics and schizotypy in an attempt to provide a suitable answer to this question.

First, it is necessary to distinguish two pairs of entities: on the one hand, although often used pseudo-synonymously, schizophrenia and psychosis are not the same. Schizophrenia is a psychotic *disorder*; featuring psychosis as a primary phenomenon, but being defined furthermore by having *Krankheitswert*. It has been shown repeatedly that (extremely) high schizotypic features and/or psychotic experiences are not limited to the clinical domain and, contrapositively, that psychotic experiences are found with considerably higher prevalence within the population [between 30 and 70% (10, 11)] than possible, if existing only in patient groups. Furthermore, it has been convincingly argued [review in Ref. (12)] that individuals, christened “*happy schizotypes*” (13), with considerably high positive schizotypy and often repeated psychotic experiences but significantly lower negative/disorganized schizotypic features (compared to the population!) are not only “not sick” but actually benefit from these experiences and are “more healthy.”

The second necessary distinction is between positive and negative/disorganized schizotypy: it is often stated that “negative schizotypy is the most heritable,” although this is better amended to “most heritable *in biological relatives of schizophrenics*” [q.v. (14)]. In the population, heritability estimates do not differ between positive and negative schizotypy, and the data suggest that the two facets are influenced genetically by two distinct latent genetic factors (15). It would, thus, seem that while positive schizotypy is the underlying dimension explaining psychotic features, it is not necessarily related to *Krankheitswert*. Negative schizotypy, however, appears closely related to schizo*phreni*a regarding its heritability as well as a major factor that differentiates “happy schizotypes” from schizophrenic patients.

Returning to endophenotypes, Gottesman and Gould (16) clearly state that configured self-report data may also constitute an endophenotype. They propose five criteria that qualify an endophenotype. I shall aim to show that schizotypy meets these criteria:

(1) Schizotypy is quite clearly associated with the clinical condition of schizophrenia. Apart from aforementioned similarities, patients with schizophrenia have repeatedly shown to have higher values in schizotypy (17), and schizotypy is elevated more highly in schizophrenic patients than in those with other psychotic disorders (18).
(2) Schizotypy is heritable (~50%) (15); although there are differences between individual schizotypy facets; with negative schizotypy being most highly heritable in patients’ relatives (14).
(3) Schizotypy as a stable trait with high re-test reliability does not solely manifest during acute phases of illness [9, 19]. There are, however, *intra*individual variations in acute schizotypal sensitivities, which are explained (mostly) by environmental influences, whereby *inter*individual differences in susceptibility to environmental influences is again a major factor of *trait* schizotypy (4, 19, 20). This is unsurprising when dealing with complex traits and strengthens the case for (state) schizotypy.
(4) Schizotypy co-segregates with schizophrenia; i.e., schizophrenic relatives of schizophrenic patients have higher values in (especially negative) schizotypy than healthy relatives of schizophrenic patients (q.v., criterion 1).
(5) Healthy biological relatives (especially offspring) of schizophrenic patients have repeatedly been shown to have higher values in (especially negative) schizotypy (14), and offspring of healthy but highly schizotypic individuals have greater risk of developing schizophrenia (21, 22).

It should be emphasized that especially heritability, co-segregation, and familial association are key points that differentiate between “true” endophenotypes and “mere” biomarkers or intermediate phenotypes [e.g., Ref. (23)]. The strong associations found between schizotypy and schizophrenia regarding the respective criteria 2, 4, and 5, thus, make a strong case toward the acceptance of psychometric schizotypy as an endophenotype of schizophrenia.

There are, however, a number of findings that need further discussion before a final conclusion may be drawn. (1) A number of genetic factors associated with schizotypy are no longer considered relevant in schizophrenia genetics. (2) A number of genetic factors associated with schizophrenia are not (or even inversely) associated with schizotypy.

To address these points, it is necessary to keep the aforementioned distinctions “psychosis vs. schizophrenia” and “positive vs. negative/disorganized schizotypy” in mind. In other words, it is expected that all schizophrenics are highly schizotypic, but *not* all highly schizotypic individuals are schizophrenic; both parts of this expectation hold true [q.v. (13)].

This latter observation, referred to by Claridge (24) as *benign schizotypy*, is an important point to consider with respect to the aforementioned two “critical points.” There is currently no general consensus regarding the exact nature of schizotypy in the entire population or the relation with schizophrenia genes. The Meehlian model proposes the group of schizotypes to be discrete, making up 10% of the population and defined *qua* the presence of (few) specific genetic risk factors. The more common view in schizotypy research, however, is that schizotypy is continuously distributed throughout the population and is genetically based on a multitude of genetic loci, whereof each have only small effect sizes, but these effects interact (9) and are additive (25); similar to recent conceptions of schizophrenia genetics (26, 27). This would give rise to an Eysenckian view (28) that schizophrenia be equal to extremely high schizotypy (or rather *psychoticism* in the Eysenckian model of personality) [q.v. (29)]. This view is, however, incompatible with the existence of *benign schizotypy* or *happy schizotypes*. I have, thus, suggested and discussed in more detail (3, 4) that the *fully dimensional* model of schizotypy (24) has the best fit regarding more recently shown genetic associations of both schizotypy and schizophrenia.

This model explains the discrepancies mentioned above in my “critical points”: it proposes that schizotypy is normally distributed in the population, but interacts with a second dimension; i.e., *health* or *resilience*. High schizotypy is in and of itself, thus, not equal to schizophrenia; when coinciding with low health/resilience, however, schizophrenia is the outcome. Schizophrenia may therefore be viewed upon as resulting from low resilience to the high [probably dopaminergic; q.v. (30)] strain on the nervous system conferred by (positive) schizotypy. The lack of association between individual schizotypy-related genetic variants and schizophrenia (e.g., the COMT-polymorphism rs4680) is therefore not surprising and does not disqualify schizotypy as an endophenotype. This is quite simply due to the fact that small effects of single schizotypy-related polymorphisms may disappear in case–control schizophrenia studies; under the assumption of the *fully dimensional* model.

The second critical point is also explained easily when assuming this model. Those genetic loci most strongly associated with the distinction between cases and controls cannot be associated with high schizotypy *within healthy individuals*. In fact, and this has been shown [e.g., Ref. (31, 32)], they *must* be inversely related to schizotypy or related measures (like psychosis-like experiences). In other words, should a person high in (especially positive) schizotypy additionally carry substantial numbers of alleles that distinguish between cases and controls (and, thus, probably load on the aforementioned dimension *resilience*), this person would *not* be found in a healthy sample. Furthermore, increasingly high levels of schizotypy must, in healthy individuals, coincide with increasingly lower genetic factors conveying risk for schizophrenia (i.e., with higher *resilience*).

To summarize and in conclusion, at first glance, schizotypy meets all the criteria as proposed by Gottesman and Gould (16) for endophenotypes in psychiatry. One could, thus, stop here and consider schizotypy *in general* a suitable endophenotype of schizophrenia. This might be jumping the gun, however, as, upon closer examination, there are a number of discrepancies between schizotypy and schizophrenia; especially related to genetics and the distinction between positive and negative/disorganized traits. I would, therefore, suggest (*sensu* 30) that (high) *positive* schizotypy be better described as an endophenotype of *psychosis-in-schizophrenia* (as well as in other psychiatric conditions or the otherwise healthy), while (high) *negative* schizotypy may be viewed upon as an endophenotype of *schizophrenia-in-psychosis* (i.e., tapping more into *Krankheitswert*). The latter is important, as (high) negative schizotypy is not exclusive to psychotic disorders, but also found to share considerable variance with another personality trait linked to other (e.g., affective or anxiety) disorders; i.e., Neuroticism (33). Schizo*phrenia* would, thus, be considered as the overlap of (very) high positive and negative schizo*typy*. This interaction between different aspects of personality (and inherent cognitive patterns) regarding the development of schizophrenia has been clearly shown by Gaweda and Prochwicz (34): while a cognitive bias typically associated with positive schizotypy (jumping to conclusions) did not differ between highly delusion-prone healthy controls and delusional schizophrenics, another bias strongly associated with negative schizotypy (catastrophizing) clearly differentiated both groups of patients (schizophrenics with and without delusions) from both groups of healthy controls (high and low delusion-proneness).

This makes schizotypy no less useful in schizophrenia research, however. I would state that it actually strengthens the case made by schizotypy researchers when saying that it is a useful and “**the** most influential, comprehensive psychological construct in schizophrenia research” [(35); p. S363; my emphasis]. This is quite simply due to the fact that in schizophrenia genetics it is not clear with what the identified genes are actually associated (proneness for *psychosis* or risk for *schizophrenia*; i.e., illness), whereas this is not the case in schizotypy. In other words, accepting discrete facets of schizotypy *per se* as suitable endophenotypes of *psychosis-in-schizophrenia* vs. *schizophrenia-in-psychosis* may not only help researching the (biological) basis of psychosis in eneral (including, of course, psychosis-in-schizophrenia), but also help us examine why certain (highly schizotypic) individuals develop schizophrenia while others benefit from their perceived “oddness.” Schizotypy research is, in my opinion, better suited for both aforementioned aspects of schizophrenia research than simple case–control designs both due to the possibility of differentiation between individual schizotypic facets and due to schizotypy’s naturally dimensional nature compared to the (artificial) dichotomy in the comparison between schizophrenic patients and “healthy” controls.

## Conflict of Interest Statement

The author declares that this research was conducted in the absence of any commercial or financial relationships that could be construed as a potential conflict of interest.
